# Endoscopic management of ingested toothpick resulting in duodeno-ureteric fistula

**DOI:** 10.1093/jscr/rjae214

**Published:** 2024-04-18

**Authors:** Georgia Roberts, Bartholomew McKay, Andrew Nathanson, Michael W Hii, Simon Banting

**Affiliations:** Department of Upper GI & HPB Surgery, St Vincent’s Hospital Melbourne, 41 Victoria Parade, Fitzroy, Victoria 3065, Australia; Department of Upper GI & HPB Surgery, St Vincent’s Hospital Melbourne, 41 Victoria Parade, Fitzroy, Victoria 3065, Australia; Department of Upper GI & HPB Surgery, St Vincent’s Hospital Melbourne, 41 Victoria Parade, Fitzroy, Victoria 3065, Australia; Department of Upper GI & HPB Surgery, St Vincent’s Hospital Melbourne, 41 Victoria Parade, Fitzroy, Victoria 3065, Australia; The Department of Surgery, St Vincent’s Hospital, The University of Melbourne, 41 Victoria Parade, Fitzroy, Victoria 3065, Australia; Department of Upper GI & HPB Surgery, St Vincent’s Hospital Melbourne, 41 Victoria Parade, Fitzroy, Victoria 3065, Australia; The Department of Surgery, St Vincent’s Hospital, The University of Melbourne, 41 Victoria Parade, Fitzroy, Victoria 3065, Australia

**Keywords:** endoscopy, foreign body, duodenum, renal pelvis

## Abstract

Toothpicks are commonly used but rarely ingested. Unlike most foreign bodies, if accidentally swallowed these rarely spontaneously pass. The duodenum has been reported as the most common site of toothpick foreign body lodgement in the upper gastrointestinal tract. We report the case of a 57-year-old presenting with recurrent urosepsis after non recognition of a toothpick impaction in the duodenum with fistulisation into the right renal pelvis. Endoscopic removal of the foreign body was successful in management of the urosepsis.

## Introduction

Toothpicks commonly cause gastrointestinal issues if inadvertently ingested. A systematic review of case reports by Steinbach *et al*. [1] recently noted 136 cases of toothpick ingestion reported in the medical literature. The most frequent reported site of lodgement of ingested toothpicks is the duodenum, occurring in 25% [2]. Toothpick migration is possible, with two previous reports of migration from duodenum into the right kidney [3, 4]. Endoscopic treatment is reported as successful with a mortality rate of 0% [1]. To our knowledge, this is the first report of a toothpick impacted in the duodenum with fistulization into the renal pelvis with recurrent presentations. Our case highlights complexities in diagnosis given the patient was unaware of toothpick ingestion, utility of CT imaging and success of endoscopic retrieval.

## Case report

A 57-year-old female presented with 6 months of recurrent urinary tract infections and right flank pain. She was managed with cystoscopic ureteric stent placement, as obstructive uropathy from uterine fibroids causing distal obstruction was initially felt to be the aetiology of recurrent urosepsis. Recurrent symptoms led to a hysterectomy being undertaken but this unfortunately did not resolve her symptoms.

Her medical history included Graves’ disease and metabolic dysfunction-associated fatty liver disease. Repeated urine microscopy demonstrated an elevated leukocytes and erythrocytes. Urinary cultures were positive for *Candida albicans*, *Candida glabrata*, and *Klebsiella aerogenes* – all upper gastrointestinal tract organisms. Initial blood tests demonstrated a normal white cell count of 8.9 × 10^9^/L (range 4.0–11.0 × 10^9^/L) and an elevated CRP of 120 mg/L (range < 5 mg/L).

**Figure 1:**
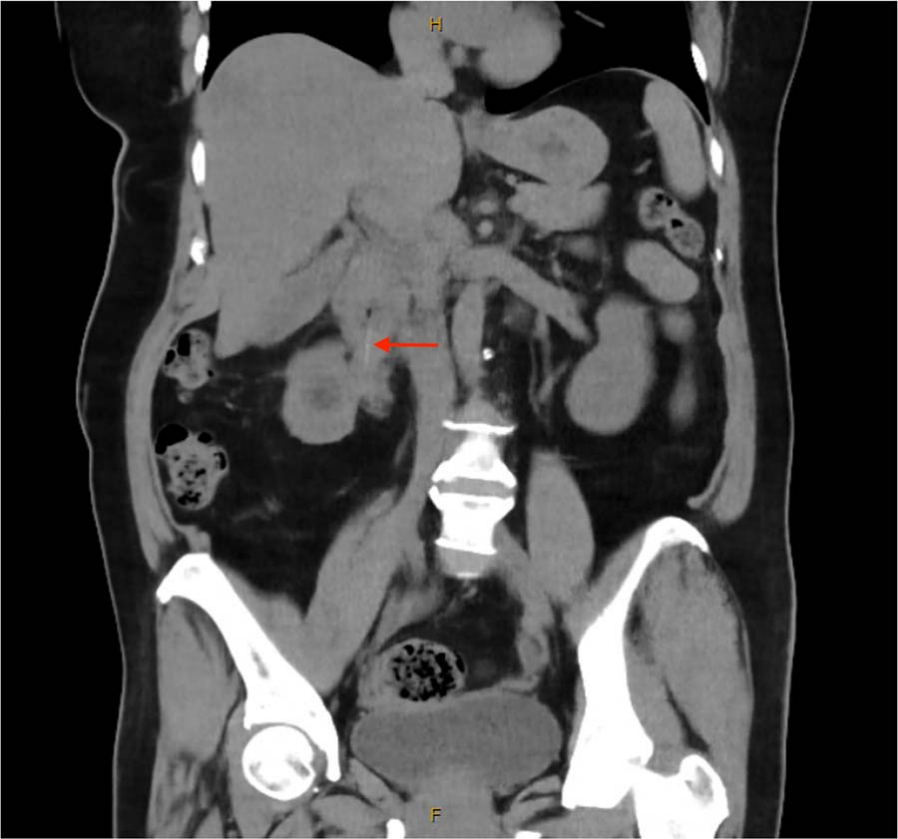
Unenhanced computed tomography of the abdomen and pelvis demonstrating a 5 cm linear radiopaque foreign body extending from the junction of the first and second part of the duodenum into the right renal pelvis.

Following ureteric stent removal, she was investigated with a routine interval computed tomography (CT) of the abdomen and pelvis (Fig. 1). This identified a 5 cm radiopaque foreign body in the proximal duodenum. The foreign body was noted to extend extra-luminally into the right renal hilum. There was right hydronephrosis proximal to foreign body, with fat stranding surrounding the right ureter. On retrospective review, this was visualized on previous CT examination. Initial attempt at endoscopic retrieval of the foreign body, which was thought to be a fish bone, was unsuccessful due to distortion of the duodenum from inflammatory changes preventing visualization. Proton-pump inhibition was commenced to reduce inflammatory changes before further attempts at retrieval.

**Figure 2:**
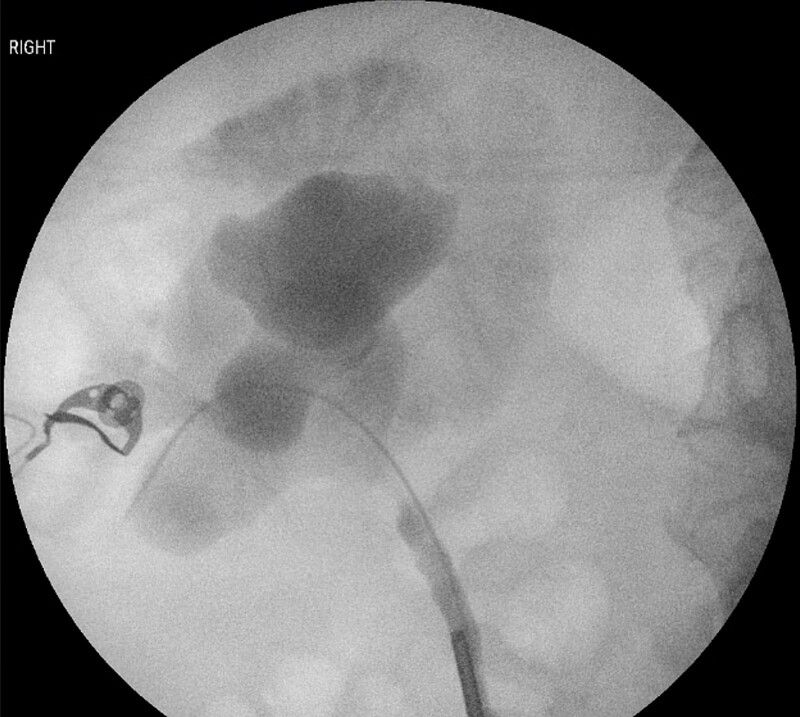
Retrograde pyelogram demonstrating contrast extravasating from the right renal pelvis into the duodenum.

Six-months after her initial presentation, she was diagnosed with recurrent urinary tract infections. Repeat CT to localize the foreign body demonstrated an unchanged position, lodged within the duodenum, extending into the right renal pelvis. A rigid ureteroscopy with a retrograde pyelogram was performed. The foreign body was unable to be visualized, however purulent fluid was egressing from the right ureteric orifice. Contrast was seen filling the renal pelvis and extravasating into the duodenum (Fig. 2). A ureteric stent was inserted for drainage.

**Figure 3:**
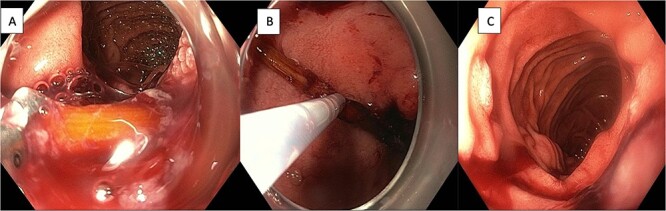
(A) Endoscopic view of toothpick lodged in the wall of the duodenum at the junction of first and second parts. (B) Endoscopic retrieval of the toothpick with through-the-scope snare. (C) Endoscopic view of second part of duodenum following retrieval of foreign body.

**Figure 4:**
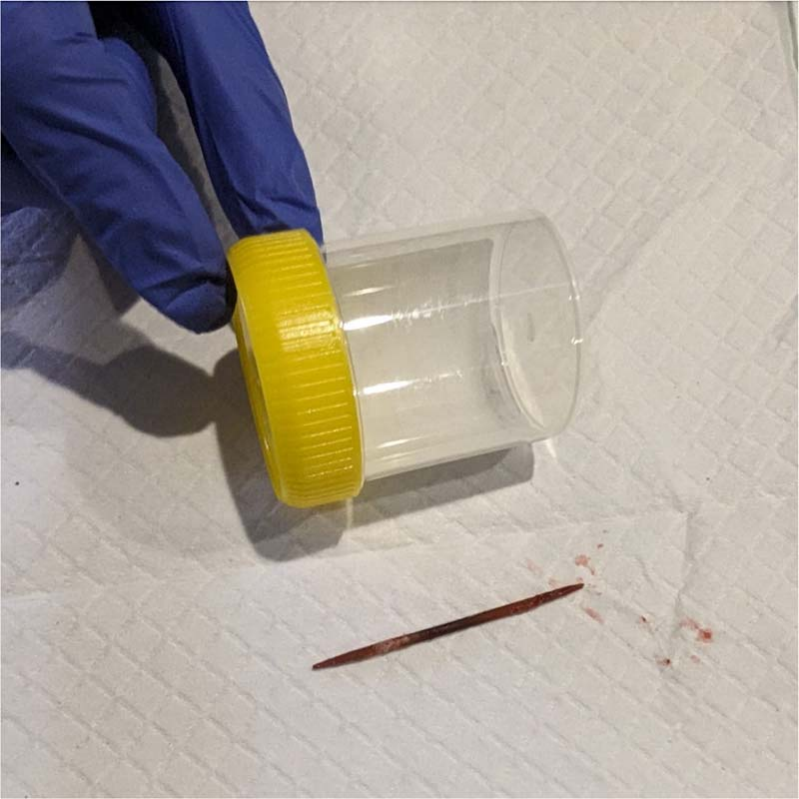
Toothpick after retrieval.

Gastroscopy one month from the previous gastroscopy, found a toothpick embedded in the duodenal wall at the junction of the first and second parts (Fig. 3A). Standard gastroscope with cap and alligator forceps was used to dislodge the foreign body into the first part of the duodenum. It was unable to be removed further with the forceps as it continued to embed in the pylorus due to the angle that it could be grasped. Additionally, with the angulation it was unable to be withdrawn into cap. Upon changing to a snare, the foreign body was successfully dislodged from the pylorus by pushing distally into the lumen before extraction by pulling the foreign body into the cap to prevent damage to the upper gastrointestinal tract (Fig. 3B and C). After it was retrieved it was confirmed to be a toothpick (Fig. 4).

The patient was questioned about their history retrospectively and had no recollection of ingestion of a toothpick. She was discharged home two days post endoscopy and remained well three months later. Serial gastroscopy one month following retrieval demonstrated a normal appearing duodenum.

## Discussion

Digestive tract foreign bodies are frequently encountered with the majority that reach the stomach passing uneventfully through the gastrointestinal tract [5, 6]. Less than 1% cause perforation, however sharp foreign bodies carry a higher risk of perforation with rates reported as high as 15%–35% [5, 6]. Toothpick ingestion is rare, with only 136 cases reported between 1927 and 2012 [1]. It commonly presents with abdominal pain localized to the area of the impacted toothpick [1]. Li and Ender [2] reported a systematic review of 57 patients with injuries from ingested toothpicks and demonstrated the most common sites of injury were the duodenum (25%) followed by the sigmoid colon (14%). Common migration sites from the duodenum include the liver, retroperitoneum, right kidney, and inferior vena cava due to their intimate relationships. If migration into the renal system occurs it can therefore lead to urinary sepsis from inoculation of the sterile urinary system with upper gastrointestinal pathogens.

On review of the literature there have been two previous case reports of toothpicks fistulating from the duodenum and into the right kidney [3, 4]. Both cases presented with haematuria and pain, whilst only one had concurrent sepsis. Endoscopic retrieval was possible in one report [3] whilst the other required laparotomy and duodenotomy to remove [4]. This is the first case that presents chronically with recurrent urinary tract infections.

Diagnosis and identification of the toothpick on imaging can be challenging. CT has been demonstrated to be more sensitive in identifying toothpicks compared with ultrasound (sensitivity 43% vs 33%, respectively) [1]. Once identified, treatment is dependent on the location of the toothpick and the clinical scenario, with options including endoscopy, laparoscopy, and laparotomy [1]. Within the duodenum, endoscopy has been reported as successful in removing toothpicks in 60% of cases [1].

The reported mortality of toothpick ingestion is high at 9%–10% [1, 2]. This is likely related to the high incidence of perforation, reported in 80% of cases of toothpick ingestion [1]. Interestingly, Steinbach *et al.* [1] reported a mortality rate of 0% in the 40 cases of toothpick ingestion managed with successful endoscopic retrieval. This supports the approach of endoscopic management in the first instance unless clinical circumstances, such as free perforation, dictate a surgical approach being required.

## Conclusion

A toothpick lodged in the duodenum has a high risk of perforation and migration. We advocate for prompt retrieval with endoscopy.
